# Comparative Multi-Omics Analysis of the Iridocorneal Angle Identifies an Immune–Fibrotic Profile in the DBA/2J Glaucoma Mouse Model

**DOI:** 10.1016/j.mcpro.2025.101499

**Published:** 2025-12-22

**Authors:** Myoung Sup Shim, Aleks Grimsrud, Vaibhav Desikan, Mi Sun Sung, Paloma B. Liton

**Affiliations:** Department of Ophthalmology, Duke University, Durham, North Carolina, USA

**Keywords:** angle region, DBA/2J, glaucoma, proteomics, RNAseq

## Abstract

We present the first integrated transcriptomic and proteomic profiling of the iridocorneal region in the spontaneous murine glaucoma model DBA/2J and DBA/2J-*Gpnmb*^+^/Sj controls to define molecular changes associated with ocular hypertension and glaucoma. Using RNA sequencing and label-free quantitative proteomics, we identified over 20,000 transcripts and 8500 proteins, creating a comprehensive molecular atlas of glaucoma-related alterations in DBA/2J mice. Principal component and differential expression analyses revealed distinct genotype-specific molecular signatures. In DBA/2J mice, upregulated genes were enriched in pathways related to extracellular matrix remodeling, collagen organization, TGF-β signaling, and inflammation. Proteomic data confirmed increased levels of complement components, antigen presentation proteins, and autophagy markers. Integrated analyses identified 29 genes upregulated at both transcript and protein levels, primarily involved in extracellular matrix structure and immune regulation. Downregulated genes were associated with melanocyte differentiation and pigment-organelle function, including *Pmel*, a gene implicated in pigmentary glaucoma. Cross-referencing with human genome-wide association studies data revealed overlap with glaucoma-associated genes (*LTBP2*, *LOXL1*, *COL11A1*, *VCAM1*), alongside reduced expression of *Angpt* and *Lmx1b*, linked to ocular hypertension. Together, these findings support the existence of an immune-fibrotic feed-forward loop and implicate collagen–elastic fiber dysfunction as a central mechanism in glaucoma pathogenesis.

## Highlights

• Integrated transcriptomic–proteomic atlas of the DBA/2J iridocorneal region.
• ECM, TGF-β, and cytokine pathways define a pro-fibrotic anterior segment state.
• Complement and autophagy networks reveal immune–fibrotic cross talk.
• LOXL1, LTBP2, ANGPT2, and LMX1B misregulated in glaucomatous angle tissue.
• GPNMB loss links ECM remodeling with innate immune activation in glaucoma.

## Graphical Abstract

**Figure:**
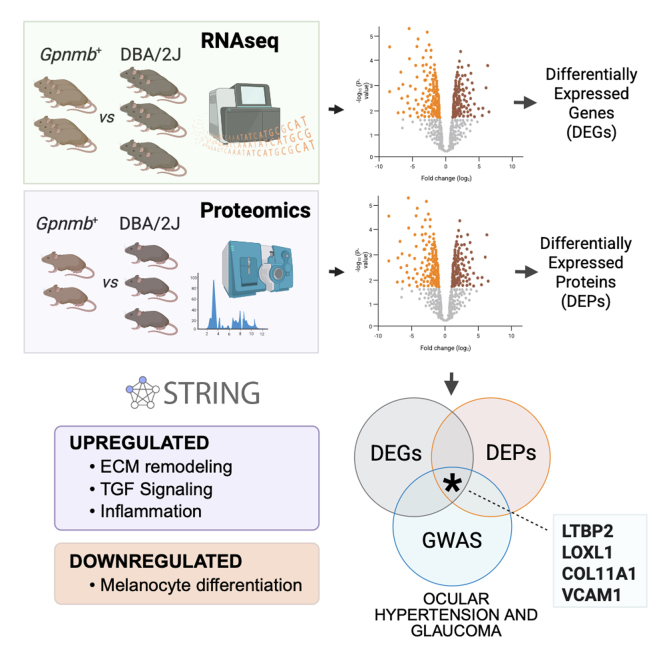

## In Brief

This study presents the first integrated transcriptomic and proteomic atlas of the iridocorneal region in DBA/2J mice, revealing coordinated activation of extracellular matrix remodeling, TGF-β, and immune pathways linked to glaucoma progression. The concordance between RNA and protein data identifies conserved risk genes and highlights an immune-fibrotic network underlying angle remodeling, providing new molecular insights into the pathogenesis of ocular hypertension and pigmentary glaucoma.

Pigmentary glaucoma is a form of secondary open-angle glaucoma that arises from pigment dispersion syndrome (PDS), a condition in which pigment granules are shed from the posterior iris pigment epithelium into the anterior chamber. These granules accumulate within the trabecular meshwork (TM), obstructing aqueous humor outflow and resulting in elevated intraocular pressure (IOP) (1). The disease predominantly affects young to middle-aged, myopic individuals, with a higher prevalence among males and those of Caucasian or European descent (2). PDS is estimated to affect approximately 1%–2.5% of the general population, and longitudinal studies indicate that between 10% and 50% of affected individuals progress to pigmentary glaucoma, depending on factors such as age, genetic susceptibility, and IOP control. The condition is typically bilateral and often asymptomatic in its early stages, making early diagnosis challenging (3, 4). While the clinical manifestations of pigmentary glaucoma are well described, the initiating defects that lead to pigment dispersion and the underlying pathophysiological mechanisms influencing progression from PDS to glaucoma remain poorly understood.

The DBA/2J mouse is a well-characterized and widely used genetic model of hereditary pigmentary glaucoma, as it recapitulates several key features of the human disease, including iris pigment dispersion, elevated IOP, and progressive optic nerve degeneration (5). The glaucomatous phenotype in this strain arises from mutations in two genes: *Gpnmb* (glycoprotein non-metastatic melanoma protein B) and *Tyrp1* (tyrosinase-related protein 1). The *Gpnmb*^R150X^ mutation leads to a loss of function in GPNMB, a transmembrane glycoprotein involved in melanosome maturation and immune regulation (6, 7), and is primarily responsible for the iris pigment dispersion phenotype (8). In parallel, the *Tyrp1*^b^ mutation disrupts melanin synthesis and melanosome stability, contributing to iris stromal atrophy, a degenerative phenotype of the iris stroma (9). These pathological changes begin to emerge between 3 and 5 months of age and become pronounced by 6 to 8 months, at which point mice typically exhibit significant pigment dispersion, accumulation of pigment-laden macrophages in the anterior chamber angle, and formation of anterior synechiae. These features collectively contribute to impaired aqueous humor outflow and elevation of IOP (5).

The DBA/2J-*Gpnmb*^+^/Sj (hereafter referred to as *Gpnmb*^*+*^) mouse strain is a genetically modified derivative of the glaucomatous DBA/2J strain, in which the wild-type *Gpnmb* allele has been restored (10). This single genetic correction markedly alters disease progression. Unlike DBA/2J mice, which carry both the *Tyrp1*^b^ and *Gpnmb*^R150X^ mutations, *Gpnmb*^*+*^ mice retain only the *Tyrp1*^b^ mutation. As a result, they exhibit the iris stromal atrophy phenotype but do not develop pigment dispersion. Importantly, *Gpnmb*^*+*^ mice do not develop IOP elevation or glaucomatous optic nerve damage, highlighting the critical and specific role of GPNMB in mediating pigment dispersion and IOP dysregulation.

While DBA/2J mice have been extensively used to investigate glaucomatous neurodegeneration (11, 12), relatively little attention has been given to the molecular alterations occurring in the iridocorneal region—where IOP dysregulation is initiated. In this study, we performed a comparative multi-omics analysis—integrating both proteomics and RNA sequencing—of the iridocorneal region in DBA/2J and *Gpnmb*^*+*^ mice to gain deeper insight into the molecular mechanisms underlying IOP elevation following iris pigment dispersion. By leveraging the genetically matched, non-glaucomatous *Gpnmb*^*+*^ strain as a control, we aimed to isolate GPNMB-specific molecular signatures associated with pigment dispersion and aqueous outflow dysfunction. Proteomics enables direct assessment of protein-level changes, including post-translational modifications and cellular signaling events, which may not be captured by transcriptomic profiling alone. Combined with RNA-seq, this approach supports high-resolution clustering and pathway analysis to identify regulatory changes involved in IOP dysregulation. Through this integrated analysis, we seek to uncover conserved molecular pathways linking pigment dispersion to IOP elevation, advancing our understanding of human pigmentary glaucoma.

## Methods

### Animal Husbandry, Genotyping and Tissue Collection

DBA/2J and *Gpnmb*^*+*^ mice were acquired from The Jackson Laboratory and bred in our facility. PCR-based genotyping was conducted on digested tail genomic DNA. The presence of the Tyrp1^b^ allele, was confirmed by amplification of a polymorphism generated by TaqI restriction site in exon 4 using *Tyrp*-F, CAGGAGCCTTCTTTCTCCCT and *Tyrp*-R, AAAGTGTCCCAGGGTATCG. Similarly, the *Gpnmb*^R150X^ mutation was confirmed by identifying the PvuII restriction site created by the mutation using *Gpnmb*-F, CTACAACTGGACTGCAGGGG and *Gpnmb*-R, AGCTCCATTTCTTCCATCCA. Mice were housed under a 12-h light/dark cycle, received a standard mouse diet, and had free access to water. Euthanasia was performed using CO_2_ asphyxiation followed by bilateral thoracotomy, after which eyes were immediately enucleated. Enucleated eyes were fixed using either 4% paraformaldehyde (PFA) for immunofluorescence studies or a mixture of 2% glutaraldehyde/2% PFA in 1 × PBS for electron microscopy, as previously described (13, 14). Tissues intended for proteomics were promptly dissected and flash-frozen on dry ice, whereas tissues designated for RNA sequencing were immediately stabilized in 0.5 ml of RNAprotect Tissue Reagent (Qiagen, 76,104) and stored at 4 °C until dissection. For dissection, the anterior and posterior chambers were separated. The anterior segment was divided into four quadrants, and the iris and ciliary body (cb) were carefully removed under a dissection microscope to avoid damaging the outflow pathway structures. Corneal and scleral tissues were trimmed and the iridocorneal angle/limbal ring (1–2 mm wide) consisting of drainage structures and some remnants of iris and cb attached was carefully dissected from each anterior segment. All experimental procedures were reviewed and approved by the Institutional Animal Care and Use Committee at Duke University (Protocol Number: A196–21–09) and conducted in accordance with the ARVO Statement for the Use of Animals in Ophthalmic and Vision Research and the National Institutes of Health Guide for the Care and Use of Laboratory Animals.

### IOP Measurements

IOP was measured using a TonoLab rebound tonometer in mice anesthetized with isoflurane. To minimize the effects of circadian fluctuations, all IOP measurements were conducted between 10:00 AM and 12:00 PM. Six consecutive readings were obtained within 1 minute of anesthesia induction and averaged to yield a single IOP value per eye per session. IOP was assessed twice monthly. After the final IOP measurement, animals were euthanized, and eyes were immediately enucleated for downstream molecular analyses. Eye enucleation and tissue procurement were performed at the same time of day to control for circadian variation across samples.

### Experimental Design and Statistical Rationale

Mice aged 12 to 13 months were used in this study. For RNA sequencing, iridocorneal angles from three individual mice (both eyes; OS/OD, six eyes total) were pooled to generate one biological replicate. Three biological replicates were prepared for DBA/2J mice (nine mice total; four females and five males) and two biological replicates for *Gpnmb*^*+*^ controls (six mice total; two females and four males). For proteomic analysis, iridocorneal angle tissue from three individual DBA/2J eyes (OS; two females and one male) and two individual *Gpnmb*^*+*^ eyes (OS; one male and one female) were independently processed and analyzed.

RNA-seq analyses were performed at the biological replicate level, where each replicate consisted of pooled iridocorneal angles from three mice, to capture inter-replicate variability and avoid pseudoreplication. Given the small sample sizes, we used methods with empirical-Bayes variance moderation to stabilize variance estimates across genes and increase precision under low n (DESeq2). Differential expression was defined using false discovery rate control and an *a priori* effect-size threshold to prioritize robust changes. For proteomics, proteins were quantified at the sample level and tested with moderated statistics suitable for small cohorts, with multiple-testing correction and emphasis on effect sizes. To increase reliability under limited power, we focused on pathway-level inferences using competitive gene set tests and rank-based enrichment, and we validate key findings by concordance between RNA and protein changes. All results are presented as exploratory and hypothesis-generating.

### RNA Extraction

Dissected iridocorneal angles from three individual mice were pooled in a 1.5 ml tube containing 100 μl of RNAprotect and stored at 4 °C until RNA extraction. RNA isolation was performed using the RNeasy Plus Micro Kit (Qiagen,74,034) according to the manufacturer’s protocol, with some modifications. Briefly, RNAprotect was removed, and tissues were lysed in 350 μl Buffer RLT Plus supplemented with 1% β-mercaptoethanol and Molecular Grinding Resin (Gbiosciences, 786,038). Tissues were homogenized using motorized pestles for 1 min and passed through a Qiashredder column (Qiagen, 79,656) by centrifugation at 13,000 rpm for 1 min. The lysate was transferred to a gDNA Eliminator spin column and centrifuged at ≥8000*g* for 30 s. An equal volume of 70% ethanol was added to the flow-through, mixed well, and the mixture was loaded onto an RNeasy MinElute spin column. After centrifugation at ≥8000*g* for 15 s, the column was sequentially washed with 700 μl Buffer RW1, 500 μl Buffer RPE, and 500 μl of 80% ethanol (repeated three times), each followed by centrifugation at ≥8000*g*. The column was then centrifuged at full speed for 5 min with the lid open to remove residual ethanol. RNA was eluted with 14 to 22 μl RNase-free water and collected by centrifugation at full speed for 1 min. RNA concentration and purity were measured using a NanoDrop spectrophotometer (DS-11^+^ spectrophotometer, DeNovix).

### RNA Sequencing and Analysis

The Sequencing and Genomics Technologies Core Facility at Duke University performed RNA-sequencing. The SGT enriched mRNA from total RNA and reversed transcribed into cDNA to build sequencing libraries using the Kapa mRNA HyperPrep Kit from Roche (Code: KK8581). Libraries were pooled to equimolar concentration and sequenced on the NovaSeq X Plus 1.5 B lane to produce 100 bp paired-end reads targeting 50M reads/sample. RNA-seq data was processed using the fastp toolkit trimming low-quality bases and sequencing adapters from the 3′ end of reads, then mapped to GRCm39 (downloaded from Ensembl, version 110) using the STAR RNA-seq alignment tool. Reads aligning to a single genomic location were summed across genes. Genes with at least 10 mapped reads in at least a single sample, were normalized and differential expression was carried out using the DESeq2 Bioconductor package implemented for the R programming environment. Consistent with the recommendation of the DESeq authors, independent filtering was utilized prior to calculating adjusted *p*-values and moderated log2 fold-changes were derived using the ashr package. Gene set enrichment analysis was performed to identify gene ontology terms and pathways associated with altered gene expression for each of the comparisons performed. Clustering analysis was performed using the Markov Clustering algorithm implemented in the STRING database to identify functionally related groups of proteins within the interaction network. Clusters were defined based on interaction confidence scores, enabling the identification of distinct protein modules potentially involved in shared biological processes.

### Differential Protein Expression Analysis by LC-MS/MS

Unbiased proteomic analysis of dissected iridocorneal region from DBA/2J and *Gpnmb*^*+*^ mice were conducted at Duke Proteomics and Metabolomics Core Facility. Samples from three individual DBA/2J eyes and two individual *Gpnmb*^*+*^ eyes were independently processed and analyzed. Tissues were lysed in 8 M urea/50 mM ammonium bicarbonate, sonicated, and protein concentration was determined by Bradford assay. Samples were normalized to 20 μg total protein, reduced with 10 mM DTT, alkylated with 20 mM iodoacetamide, acidified with phosphoric acid, and processed using S-Trap microcolumns (Protifi). Proteins were digested with sequencing-grade trypsin (20 ng/μl, 1 h at 47 °C), and peptides were sequentially eluted, dried, and reconstituted in 1% TFA/2% acetonitrile containing 12.5 fmol/μl yeast ADH as an internal standard. A study pooled quality control (SPQC) sample was created from all samples and analyzed periodically. LC-MS/MS analysis was performed on an EvoSep One system coupled to a Thermo Orbitrap Astral mass spectrometer using a DIA acquisition mode. Samples were separated using the EvoSep SPD30 gradient and analyzed with a full MS scan (m/z 380–980, resolution 240,000) followed by fixed 4 m/z DIA windows in the Astral, with HCD at 27%. Quantitative analysis was conducted using Spectronaut (Biognosys, version V20.2.250922) with a library-free Direct DIA + workflow. MS/MS spectra were searched against the SwissProt *Mus musculus* database (2024 release, containing 20,429 forward entries), a common contaminant database, and reversed decoys for false discovery rate estimation. Search parameters included fixed carbamidomethylation and variable methionine oxidation. Spectronaut employs a dynamic mass calibration strategy, with nominal starting mass tolerances of 10 ppm for precursor ions and 20 ppm for fragment ions. The false discovery rate threshold was set at 1% at both the protein and peptide levels. Razor rules were applied for protein inference, and protein grouping was based on shared peptides. Raw precursor intensities were filtered to improve quantitation reliability using multiple quality control criteria, including noise thresholding, detection frequency, signal consistency, and SPQC-based accuracy. After excluding common contaminants, data were normalized using a trimmed mean approach and missing values were imputed based on the lowest 1% of the intensity distribution. Protein-level quantification was performed using the IQ_MaxLFQ algorithm, and these normalized protein expression values were used for downstream differential expression analysis. Statistical comparisons between groups were performed using fold-change calculations and two-tailed heteroscedastic *t*-tests on log_2_-transformed protein expression data. Proteins with a fold change (FC) ≥1.5 and a *p*-value <0.05 were flagged as significantly differentially expressed. Clustering analysis was performed using the Markov Clustering algorithm implemented in the STRING database.

### Immunofluorescence on Frozen Sections

Tissue samples were fixed in 4% PFA for 24 h, then transferred to 1% PFA for long-term storage. Prior to embedding, samples underwent cryoprotection through a graded sucrose series (10%, 20%, and 30% in 1 × PBS), followed by embedding in O.C.T. compound (Tissue-Tek) and rapid freezing on dry ice. Sections were cut using a cryostat (10 μm), mounted onto charged slides, and stored at −80 °C until use. For staining, slides were equilibrated to 45 °C for 10 min, then rinsed in PBS to remove residual O.C.T. Permeabilization was performed using 0.3% Triton X-100 in PBS for 10 min at room temperature, followed by PBS washes. Nonspecific binding was blocked for 30 min at room temperature in a blocking buffer containing 5% BSA, 5% normal goat serum, and 0.3% Triton X-100 in PBS. Primary antibodies, diluted in the same buffer, were applied overnight at 4 °C. After washing, sections were incubated for 2 h at room temperature with Alexa Fluor 568-conjugated goat anti-rabbit secondary antibody (1:1000, Life Technologies) in serum-free blocking buffer. Nuclear staining was performed using DAPI (1:1000 in PBS), and slides were mounted with Vectamount AQ (Vector Laboratories, Inc) and coverslipped. Imaging was conducted using a Nikon TE2000 confocal microscope. All samples were processed and imaged under consistent conditions to minimize variability. Primary antibodies used included anti-GPNMB (1:1000, ab188222, Abcam), anti-CD31 (1:200, MAB1398Z, Millipore), and rabbit anti-LTBP2 (1:100, kindly provided by Prof. Nakamura, (15)).

### Isolation and Maintenance of Primary human trabecular meshwork Cells Culture

Primary human trabecular meshwork (HTM) cells were isolated from TM tissue obtained from discarded corneal rims following corneal transplantation at Duke University Eye Center. Each culture originated from one or two rims from the same donor and was maintained using established protocols. Cells were passaged at a 1:2 ratio in DMEM with 10% FBS and antibiotics, and passages 4 to 8 were used for experiments. HTM cells were characterized by morphology and dexamethasone-induced myocilin expression, in accordance with TM cell culture standards. All procedures were approved by the Duke IRB (protocol #00050810) and complied with the Declaration of Helsinki.

### siRNA Transfection

Primary HTM cells were seeded in 24-well plates and transfected at 80% confluence with 5 pmol of siRNA targeting GPNMB (sc-60721) using Lipofectamine RNAiMAX, following the manufacturer's protocol. A non-targeting siRNA (siCNT, sc-37007, 5 pmol) served as the control. All siRNAs were purchased from Santa Cruz Biotechnology (Dallas, TX).

### Quantitative Real-Time PCR

First-strand cDNA was synthesized from 0.5 to 1 μg of total RNA using Invitrogen Oligo(dT) primers and SuperScript II Reverse Transcriptase (Thermo Fisher Scientific), following the manufacturer’s protocol. Quantitative real-time PCR was carried out with SsoFast EvaGreen Supermix on a Bio-Rad CFX96 system using the following cycling conditions: 95 °C for 5 min, then 40 cycles of 95 °C for 5 s, 55 °C for 5 s, and 72 °C for 5 s. Ct values were calculated using the Bio-Rad CFX96 instrument software, and relative mRNA expression was determined by the comparative Ct method. Specificity of amplification was confirmed by melt curve analysis and electrophoresis on 3% Super Acryl Agarose gels. Gene expression was normalized to the average Ct values of the housekeeping gene β2-microglobulin (B2M). Primer sequences used for amplification were as follows: *LOXL1*-F: ccaggctgctatgacaccta, *LOXL1*-R: gttgcatctcaccacgttgt; *LTBP2*-F: tcctgaacggctgtgagaat, *LTBP2*-R: cgcagtaaccatggacacag, *B2M*-F: aggctatccagcgtactcca, *B2M*-R: tcaatgtcggatggatgaa, *GPNMB*-F: gttcctgacagagacccagc, *GPNMB*-R: caccaagagggagatcacagt.

## Results

### Confirmation of Loss of GPNMB Expression and Increased IOP in DBA/2J

Immunofluorescence analysis of anterior eye segments confirmed distinct differences in GPNMB expression and tissue morphology between *Gpnmb*^+^ and DBA/2J mice (Supplemental Fig. S1*A*). In *Gpnmb*^+^ eyes, GPNMB expression (magenta) was clearly localized to the TM, cb and iris. No GPNMB staining was detected in DBA/2J eyes, which exhibited disrupted angle structural integrity. These morphological alterations were accompanied by significant elevations in IOP in DBA/2J mice compared to *Gpnmb*^+^ controls over time (Supplemental Fig. S1*B*). IOP was significantly higher in DBA/2J mice at several time points, with the greatest differences observed between 8.5 to 10.0 months of age (*p* < 0.001). These findings confirmed that loss of GPNMB is associated with structural abnormalities in the anterior chamber and elevated IOP in DBA/2J mice (5, 8), a hallmark of glaucoma pathogenesis.

### Changes in mRNA Expression in the Iridocorneal Region of DBA/2J Compared to *Gpnmb*^*+*^ Mice

To investigate alterations in gene expression within the iridocorneal region of DBA/2J mice relative to *Gpnmb*^*+*^ controls, we performed RNA-seq analysis (GEO Repository accession number: GSE299237). For each replicate, iridocorneal angles from three individual mice were pooled, resulting in three biological replicates for the DBA/2J group and two replicates for the *Gpnmb*^*+*^ group. Prior to differential expression analysis, we evaluated data quality and sample-level variability using dispersion estimates and principal component analysis (Supplemental Fig. S2*A*). Dispersion values were calculated to account for gene-specific variability beyond mean expression, as required for accurate modeling with the negative binomial distribution. The dispersion estimates showed an appropriate relationship between mean expression and variability, supporting reliable downstream differential expression analysis. Principal component analysis revealed clear clustering of samples by genotype. DBA/2 J and *Gpnmb*^*+*^ samples separated along the first principal component, indicating genotype-specific transcriptomic profiles (Supplemental Fig. S2*B*).

**Fig. 1:**
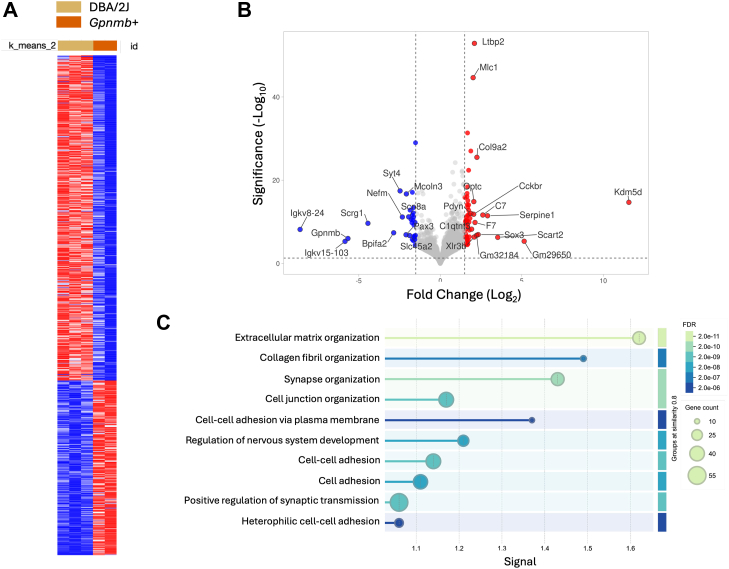
**Differential gene expression and functional enrichment analysis of the iridocorneal region in DBA/2J and *Gpnmb*^*+*^ mice.***A*, heatmap of the 709 differentially expressed genes (FC ≥1.5, *p* ≤ 0.05) between DBA/2J and *Gpnmb*^*+*^ mice, showing clear genotype-specific clustering. *Red* indicates higher expression and blue lower expression in DBA/2J mice. *B*, volcano plot of all expressed genes. Log_2_ FC is plotted on the x-axis, and –log_10_*p*-value on the y-axis. Gene’s upregulated in DBA/2J mice are shown in *red*, those downregulated in blue, and non-significant genes in *gray*. The top 25 most significantly dysregulated genes are labeled. *C*, gene ontology (GO) enrichment analysis of upregulated differentially expressed genes in DBA/2J mice, identifying significantly enriched biological processes. Pathways related to extracellular matrix organization, collagen fibril formation, and cell adhesion is highly represented. Circle size reflects gene count per pathway; color intensity reflects adjusted false discovery rate. FC, fold change; GO, gene ontology.

Across all samples, a total of 20,284 gene counts were identified (Supplemental Table S1). Differential gene expression analysis of normalized counts revealed a total of 703 genes significantly dysregulated in DBA/2J mice compared to *Gpnmb*^*+*^, using a threshold of ≥1.5-FC and *p*-value ≤0.05 (Supplemental Table S2). Among these, 456 genes were significantly upregulated, while 247 genes were downregulated in the DBA/2J group. To examine expression patterns across samples, a heat map of all differentially expressed genes (DEGs) was generated; demonstrating distinct clustering of DBA/2J and *Gpnmb*^*+*^ samples based on their transcriptomic profiles (Fig. 1*A*). To visualize global patterns of differential expression, we generated a volcano plot (Fig. 1*B*). Genes that passed significance thresholds are highlighted and the top 25 most DEGs are annotated. The complete list of DEGs is included in Supplemental Table S2.

### Hierarchical Clustering of Differentially Expressed Genes

**Fig. 2:**
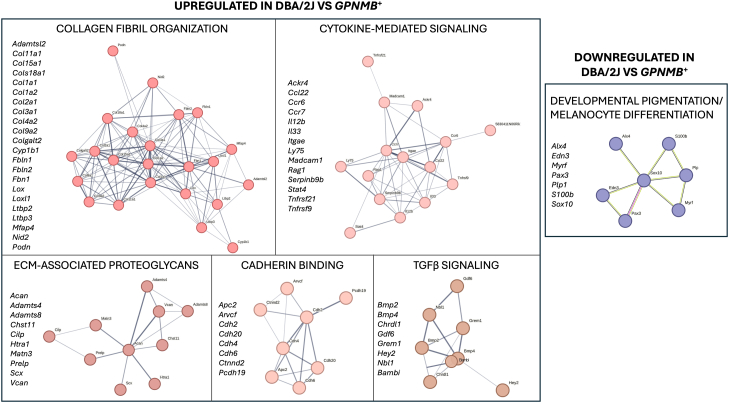
**Functional clustering of differentially expressed genes in the iridocorneal region of DBA/2J *versus Gpnmb*^+^ mice.** Differentially expressed genes identified by RNA-seq were grouped into functional categories using GO enrichment and STRING network analysis. The *left panels* represent biological processes enriched among genes upregulated in DBA/2J mice; the *right panel* shows processes enriched among genes downregulated in DBA/2J mice relative to *Gpnmb*^*+*^ controls. GO, gene ontology.

To identify functionally related groups within the DEGs, we performed gene ontology (GO) enrichment and Markov clustering (MLC) analysis using the STRING database. Figure 1*C* highlights the biological processes enriched among genes upregulated in DBA/2J mice, with prominent enrichment in extracellular matrix (ECM) organization and collagen fibril formation. Clustering analysis further revealed several significantly enriched functional groups, including collagen fibril organization, cytokine-mediated signaling, ECM-associated proteoglycans, cadherin binding, and TGFβ signaling pathways (Fig. 2). Supplemental Table S3 lists the genes with higher transcriptional expression in DBA/2J mice compared to *Gpnmb*^*+*^ in each of the top significant clusters. Developmental pigmentation and melanocyte differentiation was identified as the most significantly enriched cluster in *Gpnmb*^*+*^ compared to DBA/2J mice (Fig. 2, Supplemental Table S3).

### Comparative Analysis of Protein Content in the Iridocorneal Region Between DBA/2J and *Gpnmb*^*+*^ Mice

**Fig. 3:**
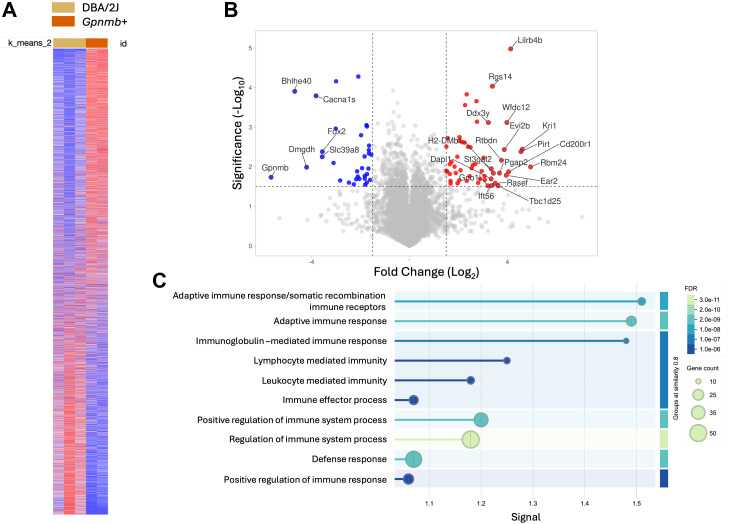
**Differential protein expression and functional enrichment analysis in the iridocorneal region of DBA/2J *versus Gpnmb*^+^ mice.***A*, heatmap of differentially abundant proteins (FC ≥1.5, *p* ≤ 0.05) between DBA/2J and *Gpnmb*^*+*^ mice, showing clear separation between groups. *Red* indicates higher abundance and *blue* indicates lower abundance in DBA/2J samples. *B*, volcano plot showing log_2_ FC *versus* –log_10_*p*-value for all quantified proteins. Proteins significantly more abundant in DBA/2J mice are shown in *red*; those less abundant are in *blue*. Selected top differentially expressed proteins are labeled. *C*, GO enrichment analysis of proteins upregulated in DBA/2J mice, highlighting biological processes primarily related to immune activation. Enriched categories include adaptive immune response, immunoglobulin-mediated responses, and leukocyte-mediated immunity. *Dot* size indicates gene count per pathway, and color represents adjusted false discovery rate. FC, fold change; GO, gene ontology.

We conducted high-resolution non-targeted quantitative proteomic analysis of dissected iridocorneal region from DBA/2J and *Gpnmb*^*+*^ mice. Samples from three individual DBA/2J mice and two *Gpnmb*^*+*^ mice were independently processed and analyzed. Using a library-free Spectronaut search with a 1% peptide false discovery rate, we identified 176,911 precursors corresponding to 8554 proteins (Supplemental Tables S4 and S5). Technical reproducibility, assessed by the average coefficient of variation (%CV) within the SPQC group, was ∼18.3%, consistent with expected LC-MS variability. Biological groups showed slightly higher variation (∼19.0%), as anticipated. Internal controls (yeast ADH and bovine casein) confirmed stable digestion and consistent technical performance. Normalized protein abundance data and group-wise comparative analyses are provided in Supplemental Table S6. A hierarchical clustering heatmap (Fig. 3*A*) illustrates expression patterns and potential group-specific signatures distinguishing DBA/2J from *Gpnmb*^+^.

Comparative analysis of protein content revealed a total of 430 differentially expressed proteins (DEPs, FC ≥1.5X, *p* < 0.05) in the iridocorneal region of DBA/2J compared to *Gpnmb*^+^. Among these, 210 proteins were more abundant and 220 were less abundant in DBA/2J. A volcano plot highlighting the top 25 differentially expressed proteins is shown in Figure 3*B*. As expected, GPNMB was found to be the less abundant protein in DBA/2J. The complete list of DEPs is included in Supplemental Table S7.

### Hierarchical Clustering of Differentially Expressed Proteins

**Fig 4:**
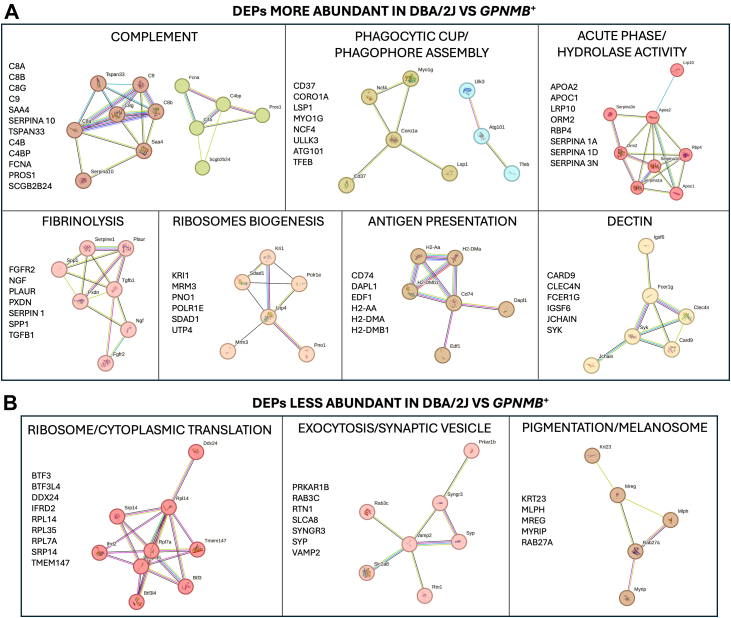
**Functional clustering of differentially expressed proteins (DEPs) in the iridocorneal region of DBA/2J *versus Gpnmb*^+^ mice.** Differentially abundant proteins identified by label-free quantitative proteomics were functionally clustered using GO enrichment and STRING protein–protein interaction network analysis. *A*, most significant networks enriched in DBA/2J mice; *B*, most significant networks enriched in *Gpnmb*^*+*^. GO, gene ontology.

We performed GO enrichment and MLC analysis of the DEPs using the STRING database. Figure 3*C* shows the enriched biological processes associated with the most differentially abundant proteins in DBA/2J mice. Notably, all identified processes were related to immune function and response, suggesting a prominent immune component in the iridocorneal region of DBA/2J mice. Clustering analysis revealed several significantly enriched functional groups, including acute phase/hydrolase activity, fibrinolysis, complement activation, ribosome biogenesis, antigen presentation, dectin signaling, and phagocytosis/autophagy (Fig. 4*A*). The lists of the proteins within the top MLC clusters enriched in DBA/2J compared to *Gpnmb*^*+*^ are included in Supplemental Table S8.

DEPs more abundant in *Gpnmb*^*+*^ mice were enriched for GO terms associated with plasma membrane proteins. Further clustering analysis identified ribosome/cytoplasmic translation, exocytosis, and pigmentation/melanosome pathways as the most underrepresented functional categories in DBA/2J mice (Fig. 4*B*, Supplemental Table S8).

### Integrative Transcriptome and Proteomic Analyses

**Fig. 5:**
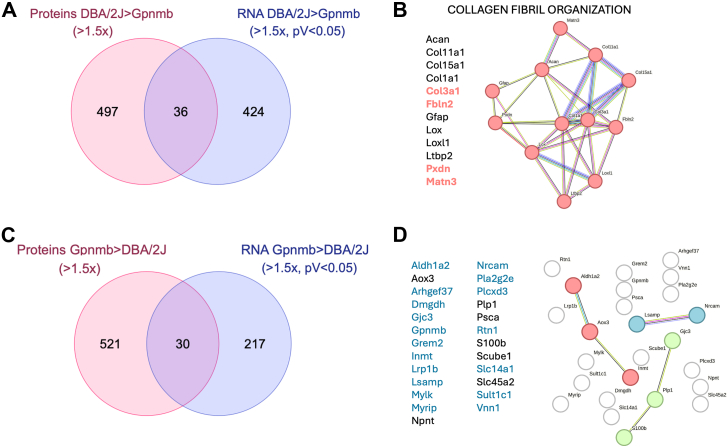
**Integrated comparative transcriptomic and proteomic analysis of DBA/2J *versus Gpnmb*^+^ mice.***A*, venn diagram showing the overlap between significantly upregulated transcripts (RNA-seq, FC >1.5, *p* < 0.05) and most abundant proteins (proteomics, FC >1.5) in DBA/2J compared to *Gpnmb*^+^ mice. *B*, venn diagram showing the overlap between significantly upregulated genes abundant proteins in *Gpnmb*^+^ compared to DBA/2J mice. *C*, STRING network analysis of concordantly upregulated genes and proteins in DBA/2J mice. Genes shown in *red* represent targets significantly upregulated at both the transcript and protein level. *D*, STRING network of genes and proteins more abundant in *Gpnmb*^+^ mice. *Blue* labels indicate genes with significant downregulation at both transcript and protein levels in DBA/2J mice. FC, fold change.

Next, we compared the DEGs with the DEPs. A total of 15 genes consistently upregulated at both the mRNA and protein levels (x-fold>1.5, pV<0.05) were identified, including *Col3a1, Dapl1, Dxd3y, Enpp6, Esm1, Fbln2, Fto, Grem1, Matn3, Plaur, Pxdn, Rbm24, Selp, Serpina3g,* and *Serpine1* [Table 1, marked *(1)* in notes column]. Considering the smaller sample size in the proteomic dataset for *Gpnmb*^*+*^, which could impose more stringent *p*-value thresholds, we conducted a secondary analysis. Here, we compared significantly upregulated mRNAs (x-fold>1.5, pV<0.05) with the full list of proteins that were increased by more than 1.5-fold in DBA/2J compared to *Gpnmb*^*+*^ mice, regardless of statistical significance. This approach identified an additional 21 proteins (Fig. 5*A*, Table 1). Among these, we highlighted 14 genes for which all individual protein normalized expression values in DBA/2J mice were higher than those in *Gpnmb*^*+*^ mice [Table 1, marked *(2)* in notes column]. These include *Acan, C3, Col11a1, Col15a1, Col1a1, F10, Gfap, Loxl1, Ltbp2, Ly75, Optc, Pipox, Stat4,* and *Vcam1*. MLC analysis of these 29 overlapping targets (Table 1), revealed enrichment in collagen fibril organization and serine-endopeptidase/complement complex (Fig. 5*B*) in the iridocorneal region of DBA/2J compared to *Gpnmb*^*+*^ mice.

**Table 1:** List of genes and proteins commonly upregulated in DBA/2J compared to Gpnmb+

Gene	Description	Proteomics						RNAseq				
		Peptides	D2 mean	Gpnmb mean	D2 vs Gpnmb (FC)	Log2FC (D2 vs Gpnmb)	pV	D2 Mean	Gpnmb Mean	Log2 FC (DBA/2J vs Gpnmb)	pV	Notes
Acan	Aggrecan core protein	4	233	141	1.66	0.73	0.09	21	5	0.72	0.002	(2)
C3	Complement C3	214	1,265,836	635,200	1.99	0.99	0.14	43,278	10,743	1.77	0.000	(2)
Clec4e	C-type lectin domain family 4 member E	1	66	25	2.63	1.39	0.36	490	172	1.00	0.000	
Col11a1	Collagen alpha-1 (XI) chain	19	80,910	35,405	2.29	1.19	0.08	5874	2923	0.93	0.000	(2)
Col15a1	Collagen alpha-1 (XV) chain	37	151,237	91,286	1.66	0.73	0.12	2814	1330	0.98	0.000	(2)
Col1a1	Collagen alpha-1 (I) chain	50	13,115,764	6,087,752	2.15	1.11	0.17	55,317	27,753	0.88	0.000	(2)
Col3a1	Collagen alpha-1 (III) chain	27	56,711	21,665	2.62	1.39	0.03	62,607	28,686	0.74	0.001	(1)
Crabp2	Cellular retinoic acid-binding protein 2	12	11,127	6579	1.69	0.76	0.27	718	326	0.72	0.001	
Crhbp	Corticotropin-releasing factor-binding protein	3	1105	660	1.67	0.74	0.25	9184	2549	1.56	0.000	
Dapl1	Death-associated protein-like 1	1	196	19	10.09	3.33	0.00	5372	2798	0.90	0.000	(1)
Ddx3y	ATP-dependent RNA helicase DDX3Y	7	3397	363	9.35	3.23	0.00	3341	2	10.61	0.000	(1)
Enpp6	Glycerophosphocholine cholinephosphodiesterase ENPP6	3	643	110	5.82	2.54	0.00	448	169	1.29	0.000	(1)
Esm1	Endothelial cell-specific molecule 1	1	63	16	4.00	2.00	0.05	2245	664	1.49	0.000	(1)
F10	Coagulation factor X	15	29,674	10,825	2.74	1.45	0.10	310	138	0.94	0.000	(2)
Fbln2	Fibulin-2	49	121,318	45,911	2.64	1.40	0.05	5679	2231	1.21	0.000	(1)
Fto	Alpha-ketoglutarate-dependent dioxygenase FTO	14	9490	5038	1.88	0.91	0.01	5505	2252	0.78	0.001	(1)
Gfap	Glial fibrillary acidic protein	46	146,303	59,246	2.47	1.30	0.09	816	381	0.92	0.000	(2)
Grem1	Gremlin-1	7	3960	693	5.71	2.51	0.03	1958	241	1.20	0.000	(1)
Ildr2	Immunoglobulin-like domain-containing receptor 2	3	826	357	2.32	1.21	0.17	7450	3078	1.15	0.000	
Lox	Protein-lysine 6-oxidase	14	51,323	18,659	2.75	1.46	0.25	7431	3044	1.05	0.000	
Loxl1	Lysyl oxidase homolog 1	26	91,169	53,543	1.70	0.77	0.10	9505	5307	0.80	0.000	(2)
Ltbp2	Latent-transforming growth factor beta-binding protein 2	45	81,616	27,718	2.94	1.56	0.12	21,849	4932	2.09	0.000	(2)
Ly75	Lymphocyte antigen 75	22	9974	4417	2.26	1.18	0.06	1042	467	1.03	0.000	(2)
Matn3	Matrilin-3	3	727	232	3.14	1.65	0.00	1829	898	0.79	0.000	(1)
Megf6	Multiple epidermal growth factor-like domains protein 6	24	10,039	6365	1.58	0.66	0.25	1453	704	0.86	0.000	
Optc	Opticin	19	40,725	12,109	3.36	1.75	0.07	28,366	5863	2.06	0.000	(2)
Pipox	Peroxisomal sarcosine oxidase	5	2670	1406	1.90	0.93	0.08	303	107	1.35	0.000	(2)
Plaur	Urokinase plasminogen activator surface receptor	5	3902	2400	1.63	0.70	0.02	800	411	0.75	0.000	(1)
Pxdn	Peroxidasin homolog	26	10,095	5913	1.71	0.77	0.01	3289	1875	0.67	0.000	(1)
Rbm24	RNA-binding protein 24	2	1835	60	30.83	4.95	0.00	495	278	0.74	0.000	(1)
Selp	P-selectin	3	982	596	1.65	0.72	0.02	1642	642	0.69	0.002	(1)
Serpina3g	Serine protease inhibitor A3G	13	33,423	12,503	2.67	1.42	0.03	51	14	1.40	0.000	(1)
Serpine1	Plasminogen activator inhibitor 1	26	31,777	12,021	2.64	1.40	0.05	27,079	2485	2.90	0.000	(1)
Sned1	Sushi, nidogen and EGF-like domain-containing protein 1	29	26,358	15,752	1.67	0.74	0.25	15,013	7053	0.80	0.000	
Stat4	Signal transducer and activator of transcription 4	1	151	24	6.30	2.66	0.14	99	43	0.81	0.001	(2)
Vcam1	Vascular cell adhesion protein 1	50	142,127	90,537	1.57	0.65	0.09	12,958	7292	0.74	0.000	(2)

For each gene, the table lists protein abundance across biological replicates, mean values; fold changes (FC), and statistical significance (pV) from proteomics, alongside corresponding RNA expression levels and differential expression metrics. (1) indicates genes that are upregulated at both the protein and transcript levels; (2) are proteins for which all individual protein normalized expression values in DBA/2J mice were higher than those in Gpnmb+ mice.

We repeated the analysis using the downregulated genes and less abundant proteins. A comparison of significantly downregulated mRNAs (FC >1.5, *p* < 0.05) with the full list of proteins decreased by more than 1.5-fold in DBA/2J mice compared to *Gpnmb*^*+*^ mice identified a total of 30 genes (Fig. 5*C*). Of these, 18 genes were consistently downregulated in DBA/2J mice at both the mRNA and protein levels (FC >1.5, *p* < 0.05), including *Aldh1a2, Arhgef37, Dmgdh, Gjc3, Gpnmb, Grem2, Inmt, Lrp1b, Lsamp, Mylk, Myrip, Nrcam, Pla2g2a, Plxnc3, Rtn1, Slc14a1, Sult1c1*, and *Vnn1* [Table 2, marked *(1)* in notes column]. An additional 7 genes—*Aox3, Npnt, Plp1, Psca, S100b, Scube1*, and *Slc45a2*—were significantly downregulated at the mRNA level, with individual normalized protein expression values consistently lower in all DBA/2J mice compared to *Gpnmb*^*+*^ controls [Table 2, marked *(2)* in notes column]. Cluster analysis of these 25 genes did not reveal any major enrichment in functional pathways (Fig. 5*D*).

**Table 2:** List of genes and proteins commonly downregulated in DBA/2J compared to Gpnmb+

Geneene	Description	Proteomics						RNAseq				
		Peptides	D2 mean	Gpnmb mean	D2 vs Gpnmb (FC)	Log2FC (D2 vs Gpnmb)	pV	D2 Mean	Gpnmb Mean	Log2 FC (DBA/2J vs Gpnmb)	pV	Notes
Aldh1a2	Retinal dehydrogenase 2	37	177,727	436,491	0.41	−1.30	0.01	1329	2596	−0.75	0.000	(1)
Aox3	Aldehyde oxidase 3	11	16,023	27,398	0.58	−0.77	0.08	299	589	−0.66	0.001	(2)
Arhgef37	Rho guanine nucleotide exchange factor 37	5	578	1499	0.39	−1.37	0.04	419	843	−0.84	0.000	
Cldn10	Claudin-10	1	142	232	0.61	−0.70	0.27	253	939	−1.71	0.000	
Dmgdh	Dimethylglycine dehydrogenase, mitochondrial	1	21	385	0.05	−4.20	0.00	28	65	−0.68	0.002	(1)
Gjc3	Gap junction gamma-3 protein	4	538	1169	0.46	−1.12	0.00	69	137	−0.63	0.001	(1)
Gpnmb	Transmembrane glycoprotein NMB	20	29,563	1,484,294	0.02	−5.65	0.00	2219	112,802	−5.67	0.000	(1)
Grem2	Gremlin-2	5	768	3488	0.22	−2.18	0.00	651	1045	−0.62	0.000	(1)
Inmt	Indolethylamine N-methyltransferase	20	34,440	104,864	0.33	−1.61	0.03	670	1998	−1.08	0.000	(1)
Itga8	Integrin alpha-8	12	2550	6067	0.42	−1.25	0.19	777	1587	−0.64	0.001	
KRT78	Keratin, type II cytoskeletal 78	16	4612	8001	0.58	−0.79	0.21	1815	3323	−0.62	0.001	
Lrp1b	Low-density lipoprotein receptor-related protein 1B	5	6332	11,565	0.55	−0.87	0.03	46	122	−0.94	0.000	(1)
Lsamp	Limbic system-associated membrane protein	11	44,710	78,172	0.57	−0.81	0.04	1036	2738	−1.22	0.000	(1)
Ly6a	Lymphocyte antigen 6A-2/6E-1	2	933	1464	0.64	−0.65	0.42	18,039	27,774	−0.58	0.000	
Mylk	Myosin light chain kinase, smooth muscle	118	259,721	413,856	0.63	−0.67	0.04	10,036	17,512	−0.68	0.000	(1)
Myrip	Rab effector MyRIP	4	236	1316	0.18	−2.48	0.02	501	1060	−0.58	0.002	(1)
Npnt	Nephronectin	10	9948	16,823	0.59	−0.76	0.20	1895	3448	−0.74	0.000	(2)
Nrcam	Neuronal cell adhesion molecule	19	4661	7523	0.62	−0.69	0.04	444	746	−0.65	0.000	(1)
Pla2g2e	Group IIE secretory phospholipase A2	3	1016	7721	0.13	−2.93	0.04	63	123	−0.63	0.001	(1)
Plcxd3	PI-PLC X domain-containing protein 3	4	958	2713	0.35	−1.50	0.01	63	235	−1.38	0.000	(1)
Plp1	Myelin proteolipid protein	6	10,085	16,056	0.63	−0.67	0.27	820	1916	−1.16	0.000	(2)
Psca	Prostate stem cell antigen	3	4527	8377	0.54	−0.89	0.14	1826	4925	−1.12	0.000	(2)
Rtn1	Reticulon-1	19	13,396	28,296	0.47	−1.08	0.01	700	1520	−0.94	0.000	(1)
S100b	Protein S100-B	1	221	651	0.34	−1.56	0.10	559	2289	−1.80	0.000	
Scube1	Signal peptide, CUB EGF-like domain-containing protein 1	14	10,214	16,073	0.64	−0.65	0.07	2470	3983	−0.62	0.000	
Slc14a1	Urea transporter 1	5	4411	8141	0.54	−0.88	0.03	290	912	−1.47	0.000	(1)
Slc45a2	Membrane-associated transporter protein	2	50	525	0.10	−3.39	0.07	149	813	−1.88	0.000	(2)
Slc4a11	Solute carrier family 4 member 11	9	1956	3807	0.51	−0.96	0.17	328	1100	−1.35	0.000	
Sult1c1	Sulfotransferase 1C1	5	154	1136	0.14	−2.88	0.00	486	1435	−1.33	0.000	(1)
Vnn1	Pantetheinase	6	1408	4930	0.29	−1.81	0.01	228	453	−0.82	0.000	(1)

For each gene, the table lists protein abundance across biological replicates, mean values, fold changes (FC), and statistical significance (pV) from proteomics, alongside corresponding RNA expression levels and differential expression metrics. Genes highlighted in blue are downregulated at both the protein and transcript levels; highlighted in grey are proteins for which all individual protein normalized expression values in DBA/2J mice were lower than those in Gpnmb+ mice.

### Identification of Glaucoma-Associated Pathways Through Integrated Proteomic and Transcriptomic Analysis

**Fig. 6:**
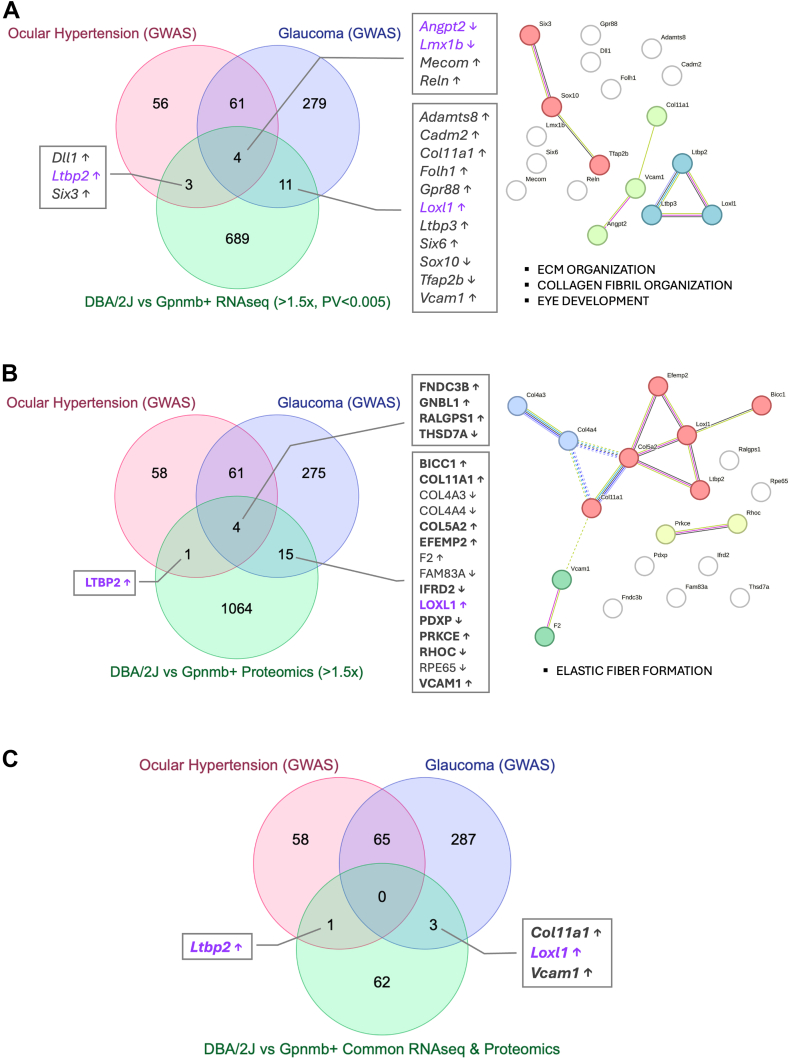
**Integration of transcriptomic and proteomic data with genome-wide association studies (GWAS) for ocular hypertension and glaucoma.***A*, venn diagram showing the overlap between differentially expressed genes in DBA/2J *versus Gpnmb*^+^ mice (RNA-seq; FC >1.5, *p* < 0.005) and loci identified in GWAS for ocular hypertension and glaucoma. The STRING network on the *right* depicts functional interactions of intersecting genes. *B*, venn diagram and corresponding STRING network analyses of overlapping more abundant proteins in DBA/2J mice (proteomics; FC >1.5) and GWAS loci for ocular hypertension and glaucoma. *C*, venn diagram showing the intersection of genes and proteins consistently differentially expressed in DBA/2J *versus Gpnmb*^+^ mice, identified by both RNA-seq and proteomics, with loci associated with ocular hypertension and glaucoma. Proteins shown in bold were either significantly altered or exhibited consistent differential expression across all DBA/2J samples. *Arrows* denote the direction of regulation in DBA/2J mice relative to *Gpnmb*^+^ controls. FC, fold change; GWAS, genome-wide association studies.

**Fig. 7:**
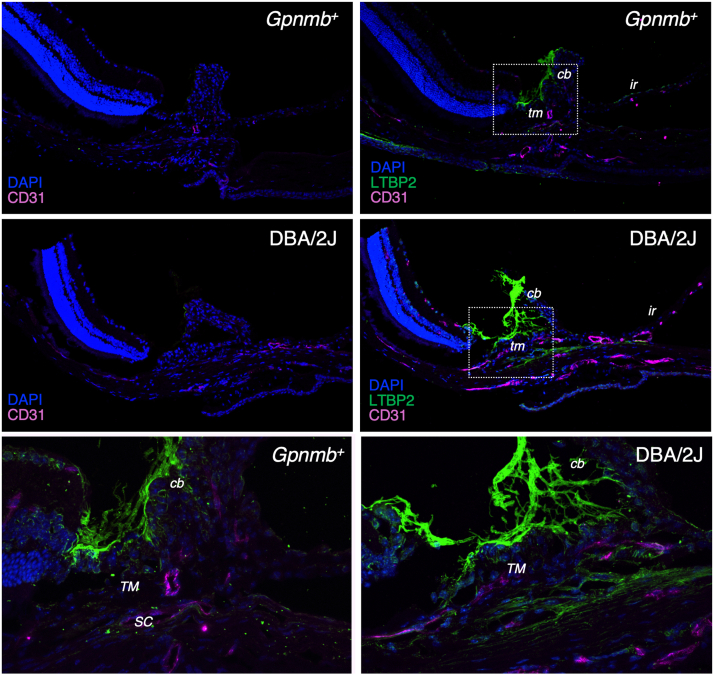
**LTBP2 expression in the iridocorneal region of *Gpnmb*^+^ and DBA/2J mice.** Representative immunofluorescence images of anterior eye segments from *Gpnmb*^+^ and DBA/2J mice (12 m.o.). Sections were stained for DAPI (*nuclei, blue*), CD31 (*endothelial marker, magenta*), and LTBP2 (*green*). *Top* and *middle panels*: Low-magnification views of the iridocorneal angle show robust LTBP2 expression in the ciliary body and in lower abundance in the trabecular meshwork. CD31 staining outlines Schlemm’s canal and vascular endothelium. *Bottom panels*: Higher magnification views of boxed regions. ir, iris.

Unlike DBA/2J mice, *Gpnmb*^*+*^ mice do not develop elevated IOP (8), providing a useful comparison for identifying regulatory changes involved in IOP dysregulation. To uncover conserved molecular pathways linking pigment dispersion to IOP elevation, we cross-referenced the DEGs and DEPs with genes previously implicated in ocular hypertension and glaucoma through genome-wide association studies (GWAS) (16). Figure 6, *A* and *B* illustrate the overlap between DEGs (FC >1.5, *p* < 0.05) and DEPs (FC >1.5), respectively, with GWAS-identified genes and enriched functional clusters. Figure 6*C* summarizes the integrative analysis of transcriptomic and proteomic data in relation to GWAS findings. Following this approach, we identified a list of genes and proteins differentially expressed in the iridocorneal region of DBA/2J mice compared to *Gpnmb*^+^, which reported association to ocular hypertension and glaucoma. Detailed descriptions of the identified genes and proteins are provided in Supplemental Table S9. Arrows next to gene or protein symbols indicate the direction of regulation in DBA/2J mice relative to *Gpnmb*^*+*^ controls. Proteins that were significantly altered or exhibited consistent differential expression across all DBA/2J mice are indicated in bold. Higher LTPB2 expression levels in the iridocorneal angle region of DBA/2J mice compared to *Gpnmb*^+^ was confirmed by immunofluorescence in frozen sections (Fig. 7, green). LTBP2 was found highly expressed in the cb and in lesser abundance in the TM region.

Cluster analysis of gene and protein expression revealed that ECM remodeling and collagen fibril organization are the most significantly enriched pathways associated with ocular hypertension and glaucoma in DBA/2J mice. The RNA-seq analysis also identified a cluster of genes involved in eye development. Four glaucoma-associated genes identified through GWAS were differentially expressed in DBA/2J mice—all showing upregulation at both the mRNA and protein levels: *Ltbp2* (latent transforming growth factor beta binding protein 2), associated with ocular hypertension; and *Loxl1* (lysyl oxidase-like 1), *Col11a1* (collagen type XI alpha 1 chain), and *Vcam1* (vascular cell adhesion molecule 1), all linked to glaucoma. Also of interest are *Angpt2* (angiopoietin 2) and *Lmx1b* (LIM homeobox transcription factor 1 beta), which were transcriptionally downregulated in DBA/2J mice according to RNA-seq analysis. Mutations in both genes are known to increase IOP in mice (17, 18, 19, 20). Unfortunately, neither was detected in the proteomic dataset.

Finally, we investigated the expression levels of genes associated with pigmentary glaucoma: *Pmel* (melanocyte protein PMEL), *Gsap* and *Grm5* (21, 22, 23). *Gsap* and *Grm5* were only detected by RNAseq; no changes in gene expression between DBA/2J and *Gpnmb*^*+*^ were observed. RNAseq showed decreased transcriptional *Pmel* (Log2FC_DBA/2J vs Gpnmb+_ = −0.29, pV = 0.015). Decreased protein levels of premelanosome protein (PMEL) in DBA/2J mice were also found by proteomics (Log2FC_DBA/2J vs Gpnmb+_ = −1,19, pV = 0.18). While it did not reach statistically significant, all the individual normalized values in DBA/2J were lower than in *Gpnmb*^*+*^ (DBA/2J: 187,836, 207,256, 164,323; *Gpnmb*^+^: 534,921, 316,498). Together, these data points toward a trend to *Pmel* downregulation in DBA/2J.

## Discussion

This study presents the first comprehensive, integrated transcriptomic, proteomic and functional cluster analyses of the iridocorneal region in DBA/2J mice and their congenic *Gpnmb*^*+*^ controls. By quantifying over 20,000 transcripts and more than 8000 proteins, we have generated a publicly accessible molecular atlas that captures key changes associated with IOP elevation in this glaucoma model. Concordant changes at the transcriptomic and proteomic levels converge on a pathogenic signature enriched for genes recently identified as risk loci in human studies of ocular hypertension and primary open-angle glaucoma (16). This overlap suggests the involvement of a conserved molecular pathway that may contribute to disease susceptibility, onset, and/or progression.

Due to the limited tissue volume, iridocorneal regions from three independent mice were pooled per replicate for RNA-seq analysis to achieve deep transcriptomic coverage without relying on amplification methods that can distort low-abundance transcripts. Any loss of biological variance introduced by pooling was mitigated by the independent, non-pooled proteomic data set that reproduced the major expression trends and stringent analytical criteria. The concordance between RNA and protein data, and the group-specific signature profiles, even with only two Gpnmb^+^ proteomes, underscores the robustness of the findings.

At the transcript level, the most prominent change is the broad upregulation of ECM remodeling genes, forming a tightly interconnected network organized into three major functional clusters: collagen fibril organization, proteoglycan components, and TGF-β signaling. This ECM signature is mirrored at the protein level, with concordant upregulation of key RNA–protein pairs including structural ECM components (ACAN, MATN3, FBLN2), crosslinking/modifying enzymes (LOXL1, PXDN, LTBP2), and regulators of proteolysis and fibrosis (PLAUR, SERPINE1), indicating coordinated activation of ECM remodeling pathways. Collectively, these molecules promote collagen cross-linking, inhibit fibrinolysis and enhance TGF-β signaling. Beyond ECM remodeling, RNA sequencing also revealed two additional significantly upregulated pathways in DBA/2J mice: cytokine-mediated signaling and cadherin binding. Together, these changes suggest a pro-fibrotic, pro-inflammatory and adhesive molecular environment within the anterior segment.

A hallmark pathological feature of DBA/2J mice is the development of anterior segment synechiae—abnormal adhesions between the iris and neighboring structures such as the cornea or TM (5). Progressive iris atrophy and pigment dispersion in these mice are thought to contribute to synechiae formation by creating a pro-inflammatory and pro-fibrotic environment within the anterior chamber (8, 9, 24, 25, 26). Inflammatory cytokines and TGF-β signaling likely drive ECM deposition and fibroblast activation, promoting adhesion formation and structural remodeling of the anterior chamber angle. Although it remains uncertain whether these adhesions are a primary pathogenic event or a secondary outcome of disease progression, the accumulation and cross-linking of ECM components likely reduce the flexibility of the iridocorneal angle, making it more susceptible to pathological adhesions.

Analysis of the protein interaction networks enriched in DBA/2J anterior segment tissues reveals a complex and coordinated activation of innate immune pathways and pro-fibrotic remodeling, highlighting the interplay between inflammation and tissue restructuring in pigmentary glaucoma pathogenesis. A prominent cluster centers on complement cascade components, reflecting robust innate immune activation at the protein level. Complement activation in the DBA/2J model has been previously implicated in driving intraocular inflammation (27). In our network, the enrichment of terminal complement proteins alongside acute-phase reactants (*e.g., Saa4*, *Serpina10*) suggests sustained humoral immune activity, likely originating from systemic sources or infiltrating macrophages—consistent with proteomic findings and histologic reports in both DBA/2J (28) and human pigmentary glaucoma (1). The higher abundance of MHC class II-associated antigen presentation machinery further points to activation of the adaptive immune system, suggesting the processing and presentation of ocular antigens by local antigen-presenting cells, likely macrophages or microglia, an event indicative of loss of ocular immune privilege. Both, innate and adaptive immune privilege have been documented to be compromised in DBA/2J mice and are believed to contribute to the progression of iris disease (25, 26, 29, 30, 31); however, they do not appear to play a direct role in intraocular pressure elevation (26).

A second prominent cluster of proteins that was selectively enriched in the iridocorneal angle of DBA/2J mice comprises components of the phagocytic and autophagic pathways. This signature most likely represents an attempt to clear the pigment granules and other cellular debris either by infiltrating immune cells or by the resident TM cells themselves (10). Particularly interesting is the marked elevation of ATG101 and ULK3—core regulators of autophagosome initiation (32)—together with the transcription factor EB (TFEB), a master controller of lysosomal biogenesis and autophagy (33). Upregulation of TFEB has been observed in a range of lysosomal storage disorders and neurodegenerative diseases, where it is thought to constitute a compensatory - and sometimes pathological-attempt to boost lysosomal degradation of toxic protein aggregates (34, 35, 36, 37). Consistent with this interpretation, our earlier work showed that DBA/2J mice exhibit diminished autophagic flux and an uncoupling between autophagosome formation and lysosomal clearance when compared with the C57BL/6J background (13). The observed up-regulation of ATG101, ULK3, and TFEB may therefore reflect a cellular effort to restore degradative capacity in the face of chronic pigment-induced stress.

Genes and proteins downregulated in DBA/2J compared to *Gpnmb*^*+*^ mice clustered around melanocyte differentiation, melanosome biogenesis, and vesicle exocytosis, consistent with the known roles of GPNMB in pigment-organelle maturation (38, 39). Of interest is the trend toward reduced PMEL expression across both molecular layers, although this decrease did not meet the stringent significance threshold in the proteomic analysis. Similar to GPNMB, PMEL is a melanosomal protein (6, 39). Non-synonymous variants in *PMEL* have been associated with ocular pigment dispersion and human pigmentary glaucoma (22), although its role in PDS remains controversial, as these findings have not been replicated in other geographic cohorts (40). Moreover, screening of *Pmel* mutant mice did not reveal a pigment dispersion phenotype (41). Regardless, these findings support a model in which defective melanosome turnover compromises pigment granule stability, increasing their susceptibility to dispersion and triggering the downstream inflammatory–fibrotic cascade described above.

In addition to *Pmel*, four genes associated with ocular hypertension and glaucoma were significantly up-regulated in the angle region of DBA/2J mice at both mRNA and protein levels: *Loxl1*, *Ltbp2*, *Col11a1*, and *Vcam1*. Interestingly, both *Loxl1* and *Ltbp2* have been linked to structural alterations in the angle region. LOXL1 plays a critical role in ECM remodeling, and its dysfunction leads to the accumulation of abnormal fibrillar material in ocular tissues (42). *LOXL1* is strongly associated with exfoliation syndrome and exfoliation glaucoma (43, 44). An association between *LOXL1* haplotypes and pigment dispersion syndrome and pigmentary glaucoma was reported by Giardina *et al* reported, suggesting a potential role in iris structural defects (45); however, this association was not confirmed in a more recent meta-analysis (46). LTBP2, another ECM glycoprotein, contributes to connective tissue organization and regulation of the TGF-β signaling pathway [(47), Torne, 2024 #1560]. Mutations in *LTBP2* have been linked to primary congenital glaucoma in both, humans (48) and cats (49), indicating its importance in eye development. To date, no association between *LTPB2* and pigmentary glaucoma has reported. The impact of *COL11A1* and *VCAM1* variants in angle structure or IOP regulation remain less well characterized.

Also of special interest is the transcriptional down-regulation of *Angpt2* and *Lmx1b* observed by RNA-seq. These transcript-level changes could not be validated in the proteomic dataset because the proteins themselves were not detected—most likely a consequence of their very low abundance in the anterior segment combined with the stringent 1% false-discovery rate threshold applied in our mass-spectrometry analysis. Angiopoietin-2 (ANGPT2) is a modulator of the Tie2 signaling axis that is essential for Schlemm’s canal maturation and maintenance (50). Murine ANGPT2 deficiency or dysregulated Angpt–Tie2 signaling produces a hypoplastic canal, impaired aqueous outflow with consequent ocular hypertension (19, 20). Likewise, the LIM-homeodomain transcription factor 1 beta (LMX1B) orchestrates early periocular mesenchyme differentiation, and heterozygous *Lmx1b* loss-of-function in mice (17, 18) or pathogenic variants in humans (*e.g.,* nail-patella syndrome) predispose to open-angle glaucoma by disrupting TM architecture and ECM turnover (51, 52).

Collectively, our integrated RNA-seq and proteomic analyses support the existence of an immune-fibrotic feed-forward loop in the pathology of DBA/2J mice, in which innate immune activation—driven by complement, recruited phagocytes, and pro-inflammatory cytokines—triggers ECM deposition and tissue remodeling. These changes are characteristic of disease-stage DBA/2J eyes and likely reflect processes active during glaucomatous progression. The specific cellular pathways through which *Gpnmb* loss contributes to these alterations remain to be defined. Interestingly, silencing *GPNMB* expression in cultured human TM cells transcriptionally downregulated *LOXL1* and *LTBP2* (Supplemental Fig. S3), suggesting that their upregulation in DBA/2J mice may occur through an indirect mechanism. GPNMB is expressed in cells from the angle region (iris, cb, TM, Supplemental Fig. S1) as well as in immune populations (26), but its functional role in either compartment is not yet defined. The observation that introducing mutant *Tyrp1* and *Gpnmb* alleles into C57BL/6J mice does not elevate intraocular pressure (53) suggests that additional DBA/2J-specific genetic modifiers are required to initiate disease. We propose here that ocular-hypertension risk genes that are misregulated in DBA/2J *(i.e., Loxl1, Ltpb2, Angpt2 and Lmx1b)* are strong candidates for such modifiers and further support the concept at collagen–elastic-fiber pathology represents a core pathogenic mechanism in ocular hypertension and glaucoma.

Interpretation of these findings should note that they reflect changes observed in diseased eyes and must account for anatomical differences in angle structure between DBA/2J and *Gpnmb*^*+*^ controls, which can confound gene expression results. For example, pigment-engulfed macrophages, which accumulate in the DBA/2J angle but are not abundant in *Gpnmb*^*+*^ eyes, may contribute to apparent upregulation of macrophage-associated genes. Similarly, synechiae formation and differences in iris structure between genotypes could lead to variable tissue representation during dissection and influence pigment-related gene expression. These structural and cellular factors likely contribute to some of the observed transcriptional differences and should be considered when interpreting the dataset. Future studies will focus on younger DBA/2J mice prior to disease progression and single cell analyses to minimize these confounding effects and clarify the earliest molecular changes associated with disease onset.

## Data Availability

RNA-seq and proteomics raw data have been deposited in the GEO and PRIDE public repositories, respectively. The GEO accession number is GSE299237; the PRIDE accession number is PXD064786. Data supporting graphs in figures is included in Supporting Data Values file.

## Supplemental data

This article contains supplemental data.

## Conflict of interest

The authors declare no competing interests.

## Supplemental Material

Supplemental Tables

Supporting Data Values

## References

[1] Alvarado J.A., Murphy C.G. (1992). Outflow obstruction in pigmentary and primary open angle glaucoma. Arch. Ophthalmol..

[2] Pang R., Labisi S.A., Wang N. (2023). Pigment dispersion syndrome and pigmentary glaucoma: overview and racial disparities. Graefes Arch. Clin. Exp. Ophthalmol..

[3] Scuderi G., Contestabile M.T., Scuderi L., Librando A., Fenicia V., Rahimi S. (2019). Pigment dispersion syndrome and pigmentary glaucoma: a review and update. Int. Ophthalmol..

[4] Bustamante-Arias A., Ruiz-Lozano R.E., Carlos Alvarez-Guzman J., Gonzalez-Godinez S., Rodriguez-Garcia A. (2021). Pigment dispersion syndrome and its implications for glaucoma. Surv. Ophthalmol..

[5] John S.W., Smith R.S., Savinova O.V., Hawes N.L., Chang B., Turnbull D. (1998). Essential iris atrophy, pigment dispersion, and glaucoma in DBA/2J mice. Invest. Ophthalmol. Vis. Sci..

[6] Chrystal P.W., Footz T., Hodges E.D., Jensen J.A., Walter M.A., Allison W.T. (2021). Functional domains and evolutionary history of the PMEL and GPNMB family proteins. Molecules.

[7] Saade M., Araujo de Souza G., Scavone C., Kinoshita P.F. (2021). The role of GPNMB in inflammation. Front. Immunol..

[8] Anderson M.G., Smith R.S., Hawes N.L., Zabaleta A., Chang B., Wiggs J.L., John S.W. (2002). Mutations in genes encoding melanosomal proteins cause pigmentary glaucoma in DBA/2J mice. Nat. Genet..

[9] Chang B., Smith R.S., Hawes N.L., Anderson M.G., Zabaleta A., Savinova O. (1999). Interacting loci cause severe iris atrophy and glaucoma in DBA/2J mice. Nat. Genet..

[10] Howell G.R., Libby R.T., Marchant J.K., Wilson L.A., Cosma I.M., Smith R.S. (2007). Absence of glaucoma in DBA/2J mice homozygous for wild-type versions of Gpnmb and Tyrp1. BMC Genet..

[11] Libby R.T., Anderson M.G., Pang I.H., Robinson Z.H., Savinova O.V., Cosma I.M. (2005). Inherited glaucoma in DBA/2J mice: pertinent disease features for studying the neurodegeneration. Vis. Neurosci..

[12] Howell G.R., Libby R.T., Jakobs T.C., Smith R.S., Phalan F.C., Barter J.W. (2007). Axons of retinal ganglion cells are insulted in the optic nerve early in DBA/2J glaucoma. J. Cell Biol..

[13] Hirt J., Porter K., Dixon A., McKinnon S., Liton P.B. (2018). Contribution of autophagy to ocular hypertension and neurodegeneration in the DBA/2J spontaneous glaucoma mouse model. Cell Death Discov..

[14] Dixon A., Shim M.S., Nettesheim A., Coyne A., Su C.-C., Gong H., Liton P.B. (2023). Autophagy deficiency protects against ocular hypertension and neurodegeneration in experimental and spontaneous glaucoma mouse models. Cell Death Dis..

[15] Inoue T., Ohbayashi T., Fujikawa Y., Yoshida H., Akama T.O., Noda K. (2014). Latent TGF-β binding protein-2 is essential for the development of ciliary zonule microfibrils. Hum. Mol. Genet..

[16] Han X., Gharahkhani P., Hamel A.R., Ong J.S., Rentería M.E., Mehta P. (2023). Large-scale multitrait genome-wide association analyses identify hundreds of glaucoma risk loci. Nat. Genet..

[17] Cross S.H., Macalinao D.G., McKie L., Rose L., Kearney A.L., Rainger J. (2014). A dominant-negative mutation of mouse Lmx1b causes glaucoma and is semi-lethal via LDB1-mediated dimerization [corrected]. PLoS Genet..

[18] Tolman N.G., Balasubramanian R., Macalinao D.G., Kearney A.L., MacNicoll K.H., Montgomery C.L. (2021). Genetic background modifies vulnerability to glaucoma-related phenotypes in Lmx1b mutant mice. Dis. Model. Mech..

[19] Kim J., Park D.Y., Bae H., Park D.Y., Kim D., Lee C.K. (2017). Impaired angiopoietin/Tie2 signaling compromises Schlemm's canal integrity and induces glaucoma. J. Clin. Invest..

[20] Schwakopf J., Romero C.O., Lopez N.N., Millar J.C., Vetter M.L., Bosco A. (2024). Schlemm's canal-selective Tie2/TEK knockdown induces sustained ocular hypertension in adult mice. Exp. Eye Res..

[21] Simcoe M.J., Shah A., Fan B., Choquet H., Weisschuh N., Waseem N.H. (2022). Genome-Wide Association Study identifies two common loci associated with pigment dispersion Syndrome/Pigmentary glaucoma and implicates myopia in its development. Ophthalmology.

[22] Lahola-Chomiak A.A., Footz T., Nguyen-Phuoc K., Neil G.J., Fan B., Allen K.F. (2019). Non-Synonymous variants in premelanosome protein (PMEL) cause ocular pigment dispersion and pigmentary glaucoma. Hum. Mol. Genet..

[23] Rong S., Yu X., Wiggs J.L. (2024). Genetic basis of pigment dispersion syndrome and pigmentary glaucoma: an update and functional insights. Genes (Basel).

[24] Anderson M.G., Smith R.S., Savinova O.V., Hawes N.L., Chang B., Zabaleta A. (2001). Genetic modification of glaucoma associated phenotypes between AKXD-28/Ty and DBA/2J mice. BMC Genet..

[25] Mo J.S., Anderson M.G., Gregory M., Smith R.S., Savinova O.V., Serreze D.V. (2003). By altering ocular immune privilege, bone marrow-derived cells pathogenically contribute to DBA/2J pigmentary glaucoma. J. Exp. Med..

[26] Anderson M.G., Nair K.S., Amonoo L.A., Mehalow A., Trantow C.M., Masli S., John S.W. (2008). GpnmbR150X allele must be present in bone marrow derived cells to mediate DBA/2J glaucoma. BMC Genet..

[27] Williams P.A., Tribble J.R., Pepper K.W., Cross S.D., Morgan B.P., Morgan J.E. (2016). Inhibition of the classical pathway of the complement cascade prevents early dendritic and synaptic degeneration in glaucoma. Mol. Neurodegener.

[28] Schraermeyer M., Schnichels S., Julien S., Heiduschka P., Bartz-Schmidt K.U., Schraermeyer U. (2009). Ultrastructural analysis of the pigment dispersion syndrome in DBA/2J mice. Graefes Arch. Clin. Exp. Ophthalmol..

[29] Nair K.S., Barbay J., Smith R.S., Masli S., John S.W. (2014). Determining immune components necessary for progression of pigment dispersing disease to glaucoma in DBA/2J mice. BMC Genet..

[30] Li Q., Pu L., Cheng S., Tang S., Zhang J., Qing G. (2024). Pigment dispersion contributes to ocular immune privilege in a DBA/2J mouse model of pigmentary glaucoma. Invest. Ophthalmol. Vis. Sci..

[31] Diemler C.A., MacLean M., Heuer S.E., Hewes A.A., Marola O.J., Libby R.T., Howell G.R. (2024). Microglia depletion leads to increased susceptibility to ocular hypertension-dependent glaucoma. Front. Aging Neurosci..

[32] Mizushima N. (2020). The ATG conjugation systems in autophagy. Curr. Opin. Cell Biol..

[33] Settembre C., Di Malta C., Polito V.A., Garcia Arencibia M., Vetrini F., Erdin S. (2011). TFEB links autophagy to lysosomal biogenesis. Science.

[34] Chen H., Gong S., Zhang H., Chen Y., Liu Y., Hao J. (2024). From the regulatory mechanism of TFEB to its therapeutic implications. Cell Death Discov..

[35] Di Malta C., Cinque L., Settembre C. (2019). Transcriptional regulation of autophagy: mechanisms and diseases. Front. Cell Dev. Biol..

[36] Cortes C.J., La Spada A.R. (2019). TFEB dysregulation as a driver of autophagy dysfunction in neurodegenerative disease: molecular mechanisms, cellular processes, and emerging therapeutic opportunities. Neurobiol. Dis..

[37] Sardiello M. (2016). Transcription factor EB: from master coordinator of lysosomal pathways to candidate therapeutic target in degenerative storage diseases. Ann. N.Y Acad. Sci..

[38] Tomihari M., Hwang S.H., Chung J.S., Cruz P.D., Ariizumi K. (2009). Gpnmb is a melanosome-associated glycoprotein that contributes to melanocyte/keratinocyte adhesion in a RGD-dependent fashion. Exp. Dermatol..

[39] Theos A.C., Watt B., Harper D.C., Janczura K.J., Theos S.C., Herman K.E., Marks M.S. (2013). The PKD domain distinguishes the trafficking and amyloidogenic properties of the pigment cell protein PMEL and its homologue GPNMB. Pigment Cell Melanoma Res..

[40] van der Heide C., Goar W., Meyer K.J., Alward W.L.M., Boese E.A., Sears N.C. (2021). Exome-based investigation of the genetic basis of human pigmentary glaucoma. BMC Genomics.

[41] Anderson M.G., Hawes N.L., Trantow C.M., Chang B., John S.W. (2008). Iris phenotypes and pigment dispersion caused by genes influencing pigmentation. Pigment Cell Melanoma Res.

[42] Zenkel M., Schlötzer-Schrehardt U. (2014). Expression and regulation of LOXL1 and elastin-related genes in eyes with exfoliation syndrome. J. Glaucoma.

[43] Thorleifsson G., Magnusson K.P., Sulem P., Walters G.B., Gudbjartsson D.F., Stefansson H. (2007). Common sequence variants in the LOXL1 gene confer susceptibility to exfoliation glaucoma. Science.

[44] Schlötzer-Schrehardt U. (2009). Molecular pathology of pseudoexfoliation syndrome/glaucoma--new insights from LOXL1 gene associations. Exp. Eye Res..

[45] Giardina E., Oddone F., Lepre T., Centofanti M., Peconi C., Tanga L. (2014). Common sequence variants in the LOXL1 gene in pigment dispersion syndrome and pigmentary glaucoma. BMC Ophthalmol..

[46] Rong S., Yu X. (2024). Lack of Association between LOXL1 variants and pigment dispersion Syndrome/Pigmentary glaucoma: a meta-analysis. Genes (Basel).

[47] Narooie-Nejad M., Paylakhi S.H., Shojaee S., Fazlali Z., Rezaei Kanavi M., Nilforushan N. (2009). Loss of function mutations in the gene encoding latent transforming growth factor beta binding protein 2, LTBP2, cause primary congenital glaucoma. Hum. Mol. Genet..

[48] Ali M., McKibbin M., Booth A., Parry D.A., Jain P., Riazuddin S.A. (2009). Null mutations in LTBP2 cause primary congenital glaucoma. Am. J. Hum. Genet..

[49] Kuehn M.H., Lipsett K.A., Menotti-Raymond M., Whitmore S.S., Scheetz T.E., David V.A. (2016). A mutation in LTBP2 causes congenital glaucoma in domestic cats (Felis catus). PLoS One.

[50] Thomson B.R., Souma T., Tompson S.W., Onay T., Kizhatil K., Siggs O.M. (2017). Angiopoietin-1 is required for Schlemm's canal development in mice and humans. J. Clin. Invest..

[51] Gardin M.A., Khor C.C., Silva L., Krefting E.A., Ritch R. (2020). Plateau iris syndrome and angle-closure glaucoma in a patient with nail-patella syndrome. Am. J. Ophthalmol. Case Rep..

[52] Pressman C.L., Chen H., Johnson R.L. (2000). LMX1B, a LIM homeodomain class transcription factor, is necessary for normal development of multiple tissues in the anterior segment of the murine eye. Genesis.

[53] Anderson M.G., Libby R.T., Mao M., Cosma I.M., Wilson L.A., Smith R.S., John S.W. (2006). Genetic context determines susceptibility to intraocular pressure elevation in a mouse pigmentary glaucoma. BMC Biol..

