# Supplementary material for: Comparative Multi-Omics Analysis of the Iridocorneal Angle Identifies an Immune–Fibrotic Profile in the DBA/2J Glaucoma Mouse Model
Source: Mol Cell Proteomics. 2025 Dec 22;25(2):101499. doi: 10.1016/j.mcpro.2025.101499 (PMC12861312; doi:10.1016/j.mcpro.2025.101499)
Supplement: Supplemental Material [file mmc1.pdf]

SUPPLEMENTAL FIGURES

Comparative Multi-Omics Analysis of the Iridocorneal Angle Identifies an Immune–Fibrotic Profile in the DBA/2J Glaucoma Mouse Model

Myoung Sup Shim, Aleks Grimsrud, Vaibhav Desikan, Mi Sun Sung, Paloma B. Liton*

Duke University, Department of Ophthalmology, Durham, NC 27705, USA

- **Supplemental Figure 1**: Confirmation of loss of GPNMB expression and IOP elevation in DBA/2J mice.
- **Supplemental Figure 2**: Data quality and sample-level variability in RNA-seq analysis of the iridocorneal region.
- **Supplemental Figure 3**: Effect of silencing Gpnmb on LOXL1 and LTBP2 expression in primary cultures of human TM cells

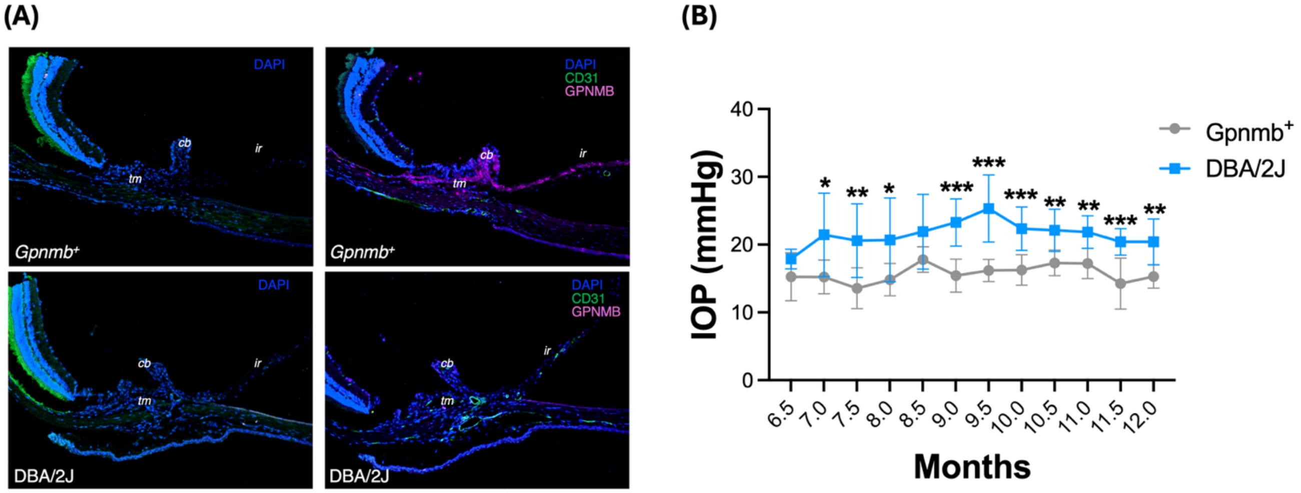

Supplemental Figure 1: Confirmation of loss of GPNMB expression and IOP elevation in DBA/2J mice. (A) Representative immunofluorescence staining of anterior eye segments from *Gpnmb* $^{+}$and DBA/2J mice. Sections were labeled with DAPI (nuclei, blue), CD31 (endothelial cells, green), and GPNMB (magenta). Panels on the left are negative controls. *Gpnmb* $^{+}$ eyes show strong GPNMB expression in the TM, and ciliary body (cb), and iris (ir). **(B)** IOP measurements over time in Gpnmb^{+} (gray) and DBA/2J (blue) mice. DBA/2J mice show significantly elevated IOP beginning at 7.0 months, peaking between 8.5–10.0 months (***$p < 0.001$, **$p < 0.01$, t-test, n=12). Data are presented as mean ± SD.

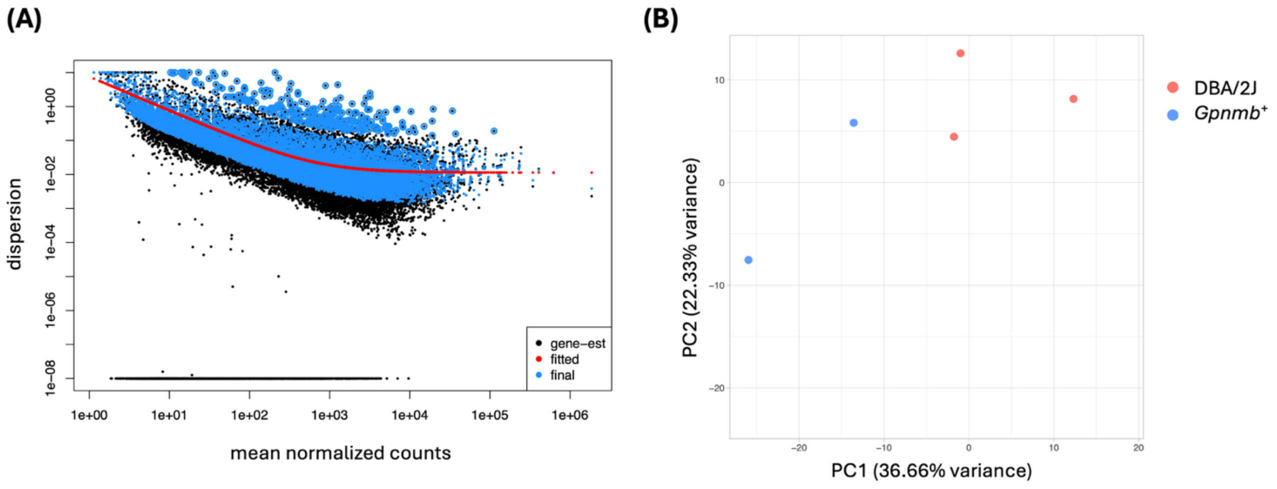

Supplemental Figure 2. Data quality and sample-level variability in RNA-seq analysis of the iridocorneal region. (A) Dispersion plot showing the relationship between mean normalized gene expression and dispersion estimates for all genes included in the differential expression analysis. Each dot represents a gene; black points indicate raw dispersion estimates, blue points indicate the final adjusted dispersions used for modeling, and the red line represents the fitted dispersion trend. The distribution supports reliable variance modeling using a negative binomial framework. **(B)** Principal component analysis (PCA) plot of normalized gene expression profiles from DBA/2J (red) and *Gpnmb* $^{+}$(blue) samples. Each point represents a pooled biological replicate (3 for DBA/2J, 2 for *Gpnmb* $^{+}$). Samples cluster by genotype along the first principal component (PC1, 36.66% variance), indicating clear transcriptomic separation between the two groups.

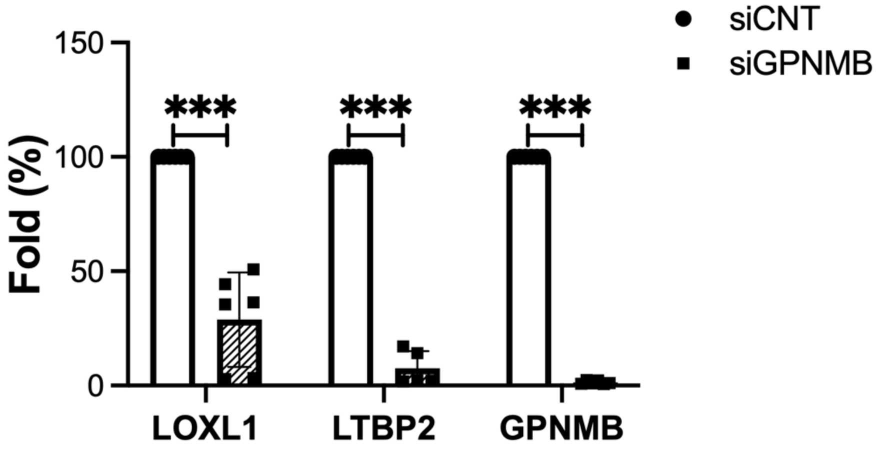

Supplemental Figure 3. **Effect of silencing Gpnmb on LOXL1 and LTBP2 expression in primary cultures of human TM cells**. TM cells were transfected with control siRNA (siCNT) or GPNMB-targeting siRNA (siGPNMB) for 3 days. Gene expression levels of LOXL1, LTBP2, and GPNMB were assessed by qPCR and normalized to control siRNA-treated cells (set as 100%). Data represent mean ± SEM from N=6 . ***P < 0.001, paired *t*-test.
